# Amelioration of the Oxidative Stress Generated by Simple or Combined Abiotic Stress through the K^+^ and Ca^2+^ Supplementation in Tomato Plants

**DOI:** 10.3390/antiox8040081

**Published:** 2019-03-30

**Authors:** María García-Martí, María Carmen Piñero, Francisco García-Sanchez, Teresa C. Mestre, María López-Delacalle, Vicente Martínez, Rosa M. Rivero

**Affiliations:** 1 Department of Plant Nutrition, Campus Universitario de Espinardo, CEBAS-CSIC, Ed 25, Espinardo, 30100 Murcia, Spain; mgmarti@cebas.csic.es (M.G.-M.); mcpinerozapata@gmail.com (M.C.P.); fgs@cebas.csic.es (F.G.-S.); tmestre@cebas.csic.es (T.C.M.); mlopez@cebas.csic.es (M.L.-D.); vicente@cebas.csic.es (V.M.); 2 Programa de Doctorado en Ciencias de la Salud, Campus de los Jerónimos, UCAM (Universidad Católica San Antonio de Murcia), s/n, 30107 Murcia, Spain

**Keywords:** abiotic stress combination, heat stress, salinity, photosynthesis, lipid peroxidation, ROS detoxification, antioxidant-related genes, antioxidant enzymes

## Abstract

Abiotic stressors such as drought, heat, or salinity are major causes of yield loss worldwide due to the oxidative burst generated under these conditions. Recent studies have revealed that plant response to a combination of different environmental stressors is unique and cannot be deduced from the response developed to each stress when applied individually. Some studies have demonstrated that a different management of some nutrients in the irrigation solution may provide an advantage to the plants against abiotic stressors. Thus, the aim of this study was to investigate if an increase in potassium (K^+^) and calcium (Ca^2+^) concentration in the nutrient solution may have a positive effect on the amelioration of oxidative stress which occurs under the combination of salinity and heat in tomato plants. Our results indicated that plants irrigated with an increase in K^+^ and Ca^2+^ concentrations in the irrigation solution from 7mM (K^+^) to 9.8 mM and from 4 mM (Ca^2+^) to 5.6 mM, respectively, induced a recovery of the biomass production compared to the plants treated with salinity or salinity + heat, and subsequently irrigated with the regular Hoagland solution. This was correlated with a better performance of all the photosynthetic parameters, a reduction in the foliar concentration of H_2_O_2_ and a lower lipid peroxidation rate, and with a better performance of the antioxidant enzymes ascorbate peroxidase ascorbate peroxidase (APX), dehydroascorbate reductactase (DHAR), glutathione reductase (GR), and NADPH oxidase. Our results showed that these enzymes were differentially regulated at the transcriptional level, showing a higher reactive oxygen species (ROS) detoxification efficiency under salinity and under the combination of salinity and heat, as compared to those plants irrigated with common Hoagland. An increase in K^+^ and Ca^2+^ in the irrigation solution also induced a lower Na^+^ accumulation in leaves and a higher K^+^/Na^+^ ratio. Thus, our study highlights the importance of the right management of the plant nutritional status and fertilization in order to counteract the deleterious effects of abiotic stress in plants.

## 1. Introduction

Tomatoes (*Solanum lycopersicum*) are the second-most important cultivar in the world economically. Southeastern agriculture in Spain is characterized by its production of high-quality agronomical products with a high added value that are commercialized in both national and international markets, resulting in large revenues for the Spanish regions dedicated to tomato cultivation. Unfortunately, adverse environmental conditions, such as salinity (coming from both low-quality irrigation water and salinized soils), scarcity of water, and high temperatures, negatively affect plant production and considerably increase economic losses. These environmental conditions are even more detrimental when they act in concert, resulting in great economic losses worldwide. On the other hand, the climate change predictions expected in the next 50–80 years, as published by the International Panel for Climate Change [1], indicate a worsening of the current environmental conditions that, together with the increase in the world’s population, make clear the urgent need for obtaining cultivars and the proper management of crops, in order to increase abiotic stress tolerance and improve yields. 

Salinity reduces crop production and negatively impacts the rate of leaf expansion, protein synthesis, energy production, lipid metabolism, opening of stomata, and photosynthesis through osmotic stress caused by soil water deficits, ion toxicity, and nutrient imbalances [2]. Similarly, heat stress affects biomass accumulation in crops of agronomic importance, such as tomato, by impairing the correct functioning of photosystem II (PSII) and reducing the chlorophyll content, resulting in a reduction in plant growth [3,4], which in turn negatively affects final fruit production. 

In the last few years, our research group has successfully demonstrated that the combination of two or more abiotic stressors generated a specific plant response that could not be elucidated from the responses obtained when these stressors were applied individually [5,6,7]. This highlights the importance of studying abiotic stressors in combination rather than separately. Individual as well as combined abiotic stressors induce imbalances in the redox cell homeostasis due to the accumulation of reactive oxygen species (ROS), such as hydrogen peroxide (H_2_O_2_) [8]. Despite their potential to cause damage, it has been shown that, at non-toxic concentrations, H_2_O_2_ plays a key role in signal transduction mechanisms, which in turn activate defense responses to various stressors [9]. 

To remove excess ROS and to maintain redox homeostasis, plants have a well-integrated antioxidant defense system that is comprised of antioxidant compounds (i.e., ascorbate, glutathione, β-carotene, etc.) and enzymes, such as superoxide dismutase (SOD), catalase (CAT), and those involved in the ascorbate-glutathione (AsA–GSH) cycle [10]. Under control conditions, these enzymes are tightly regulated for achieving a high efficiency in ROS detoxification. However, abiotic stressors cause inhibition at transcriptional and post-transcriptional levels, as well as post-translational modifications that translate into a decrease in detoxification efficiency, and thus ROS accumulation and deleterious damage to the cells [8]. 

Plant growth and metabolism are also dependent on plant nutritional status. In this sense, potassium (K^+^) and calcium (Ca^2+^) nutrition plays a fundamental role. Most biochemical and physiological processes, such as protein synthesis, regulation of plant stomata, water use, control of ionic balance, activation of enzymes, and many other processes, are affected by K^+^, positively contributing to the survival of plants exposed to various abiotic stressors [11,12]. In plants subjected to salinity stress, it is common to find a K^+^ deficiency [13,14], and a low concentration of K^+^ under saline conditions can result in the formation of ROS and cell damage [15]. Also, it has been shown that Ca^2+^ is essential for selective transport by plant cells. An extra Ca^2+^ supply may contribute to cell wall and plasma membrane integrity, and thus may reduce the effect of salinity on plants [16,17]. Moreover, it has also been shown that K^+^ nutrition improved with an application of external Ca^2+^ under salinity stress [18]. ROS have been described as playing an important role in the root’s response to nutrient deprivation. In this sense, K^+^ deprivation induced ROS accumulation in Arabidopsis [12,18,19] and tomato roots [20], with these ROS proposed to participate in the signaling cascade that results in the induction of the genes encoding for the high affinity K^+^ transporter HAK5 [12]. The study of salinity and heat stress applied individually has contributed important information to the current knowledge on abiotic stressors, but recent research studies have indicated that a plant’s response to a combination of two or more stressors is unique and cannot be elucidated from the study of single stressors [6,7,21,22,23], so stress combination studies are deemed very necessary. Thus, the aim of the present study was to investigate the beneficial effects of an increase in K^+^ and Ca^2+^ concentration in the irrigation solution on the tolerance of tomato plants to the combination of salinity and high temperature, through the amelioration that these elements can exert on the oxidative stress generated under these conditions. 

## 2. Materials and Methods

### 2.1. Preliminary Experiments on K^+^ and Ca^2+^ Doses in the Nutrient Solution

Before the main set of experiments was performed, a preliminary experiment was conducted in order to confirm the hypothesis that an increase in the concentrations of K^+^ and Ca^2+^ in the irrigation solution of tomato plants could exert a positive effect on plant biomass production under an abiotic stress combination, and more specifically, under the combination of salinity and heat, two of the most devastating abiotic stressors on plant productivity that act jointly in the southeast of Spain. For this purpose, tomato seeds (*Solanum lycopersicum* L.) cultivar “Boludo” (kindly offered by Monsanto, Torre Pacheco, Murcia, Spain) were sown in vermiculite in a growth chamber under optimal and controlled conditions of light (500 µmol m^−2^ s^−1^), photoperiod (16/8 h day/night), humidity (60–65%), and temperature (25 °C) (Chamber A, see Table 1 below). When the plants reached a height of 8–10 cm, they were transferred to 20 L containers (a total of 96 plants, two plants per container) and grown in an aerated hydroponic system containing Hoagland solution (Supplementary Table S1, [24]). The plants were grown under these conditions for 10 days in order to acclimate them to this growing system. After this time, half of the plants (48 plants) were transferred to another growth chamber with the same light, photoperiod, and humidity parameters described previously, but with the environmental (air) temperature set at 35 °C (Chamber B, see Table 1 below). At this time, the different treatments (T1, T2 and T3) were applied as follows (*n* = 12): 

Plants were kept under these conditions for 21 days, after which time the fresh weight and dry weight of whole plants were recorded (Supplementary Figure S1). Based on the results obtained in this preliminary experiment, treatment 2 (T2) (i.e., 9.8 mM K^+^ and 5.6 mM Ca^2+^) was selected for further experiments, as it was the treatment that obtained a better biomass production under the stressors applied. 

### 2.2. Experimental Design, Plant Material and Growth Conditions

Tomato seeds (*Solanum lycopersicum* L.) cv. “Boludo” were sown as described previously. When the plants were 8–10 cm in height. A total of 48 plants were transferred to 120 L tanks (with one plant per tank) and grown in an aerated hydroponic system containing the control Hoagland solution (as described in Supplementary Table S1) for 10 days in order to adapt them to this system. At this time, the plants were divided between two polycarbonate greenhouses (24 plants per greenhouse) located in an experimental station in Santomera (Murcia, Spain) during the spring growing season, and the different treatments were applied for 30 days. Salinity was applied at the same time that plants were separated into the two different greenhouses, as shown in Table 2. Thus, the experiment consisted of six treatments, as follows: 

Control (25 °C), heat stress (35 °C), salinity stress (25 °C + 60 mM NaCl), salinity combined with increases in K^+^ and Ca^2+^ concentrations (25 °C, 60 mM NaCl, 9.8 mM K^+^ and 5.6 mM Ca^2+^), salinity and heat stress combined (60 mM NaCl + 35 °C), and salinity and heat stress combined with the increases in K^+^ and Ca^2+^ concentration in the nutrient solution (60 mM NaCl, 35°C, 9.8 mM K^+^ and 5.6 mM Ca^2+^) were set as treatments. Eight tanks per treatment were randomly distributed in both greenhouses (Supplementary photograph 1). The pH of the all the nutrient solutions used in our experiments was maintained between 5.5 and 6.1. Water lost by transpiration was replaced every two days and nutrients were added every week to restore their initial concentrations. To apply the temperature treatments, this parameter was set at a maximum of 25 °C for greenhouse A (control) and 35 °C for greenhouse B. The greenhouse climate control system operated as described in Rodriguez-Ortega et al. [25]. 

### 2.3. Plant Sampling

Six plants were used per treatment. Thirty days after starting the treatments, the tomato plants were harvested and divided into leaves, stems, and roots, and their fresh weight (FW) and dry weight (DW) were recorded. Half of the harvested plant material was immediately submerged into liquid N_2_ and stored for future procedures. Fully expanded leaves corresponding to the 6th–8th position (middle leaves) were separated and used for the biochemical and molecular measurements. The other half of the plant material was oven-dried for 72 h at 70 °C and weighed to determine the dry weight (DW). Dried plant material was used for ion analysis. 

### 2.4. Ion Analysis and Quantification

Dried plant material was digested with HNO_3_:HClO_4_ (2:1, *v:v*), and Na^+^ and K^+^ concentrations were determined by atomic absorption spectrometry (Perkin-Elmer 5500, Waltham, MA, USA). 

### 2.5. Leaf Gas Exchange 

Net photosynthesis rate (A), stomatal conductance (gs), and transpiration rate (E) were measured at the end of the experiment in the youngest fully-expanded leaf of each plant (six plants per treatment), using a LI-6400XT photosynthesis system (Li-Cor, Inc., Lincoln, NE, USA). Leaf gas exchange was measured as described by Martinez et al. [6]

### 2.6. H_2_O_2_ Quantification

H_2_O_2_ was extracted as described by [26], with some modifications [23,27,28]. The concentration of H2O2 in the extracts was determined as described by Martinez et al. [6] 

### 2.7. Lipid Peroxidation

Malondialdehyde (MDA), as a degradation product of lipid peroxidation, was determined as outlined by Fu and Huang [29] with the modifications listed by Mestre et al. [24], using an MDA extinction coefficient of 155 mM^−1^ cm^−1^.

### 2.8. RNA Extraction and qRT-PCR Experiments 

Total RNA was isolated from whole tomato leaves, as described by Martinez et al. [6]. The primer sequences used for the different genes tested as well as the primer sequences of the internal controls are available in Supplementary Table S2. The normalization of transcript expression against housekeeping genes, reaction components, and PCR settings were carried out as described by Martinez et al. [6]. Expression rates as well as log_2_ values of the different genes can be found in Supplementary Table S3.

### 2.9. Enzymatic Activities 

All the enzymatic activities described were extracted as described by Martinez et al. [6]. Enzymatic activities were expressed as per mg protein [30].

CAT was assayed as described by Chance and Maehly [31]. The activity was expressed as the change in absorbance at 240 nm of a solution of 12.5 mM H_2_O_2_ in a 50 mM KH_2_PO_4_ buffer (pH 7.0) at 30 °C. CAT activity was calculated using an extinction coefficient of 39.4 mM^−1^·cm^−1^ [32]. 

Activities of the Cu/ZnSOD and FeSOD isoenzymes were assayed as described by McCord and Fridovich [33], with some modifications [34]. The reaction mixture and the quantification of Cu/ZnSDO and Fe SOD are fully described in Martinez et al. [6] 

Cytosolic APX (cAPX), monodehydroascorbate reductase (MDHAR), and dehydroascorbate reductase (DHAR) activities were assayed as described by Miyake and Asada [35] and the full method can be found in Martinez et al. [6]. 

GR activity was assayed as described previously by Mestre et al. [24], with the modification of Martinez et al. [6].

NADPH oxidase activity was measured following the method described by Kaundal et al. [36]. The NADPH oxidase-dependent generation of O_2_^−^ in plant tissue has been determined by the reduction of the tetrazolium salt 3′-{1-[(phenylamino)-carbonyl]-3,4-tetrazolium}bis(4-methoxy-6-nitro)benzenesulfonic acid hydrate (XTT) by O_2_^−^. In the presence of O_2_^−^, XTT generates a soluble yellow formazan that can be quantified spectrophotometrically at 490 nm. The rate of O_2_^−^ generation was calculated using an extinction coefficient f 2.16 × 10^4^ M^−1^ cm^−1^ [37].

Different absolute values obtained for each treatment and enzyme (Supplementary Table S4) were normalized against its control and log_2_ values were calculated (Supplementary Table S5). Enzymatic activities were represented as a heat map of these log_2_ values obtained.

### 2.10. Statistical Analysis

The data were first tested for homogeneity of variance and normality of distribution, and the Tukey HSD (Honestly-significant-difference) multiple range test was used to determine differences between means (*p* ≤ 0.05), with *p* < 0.05 *, *p* < 0.01 **, *p* < 0.001 *** and n.s. as not significant. The relative transcript expression assayed by quantitative PCR (qPCR) was calculated using the 2^−^^ΔCt^ method. Heat maps for transcript expression and enzymatic activities were created using R.

## 3. Results and Discussion

**Figure 1:**
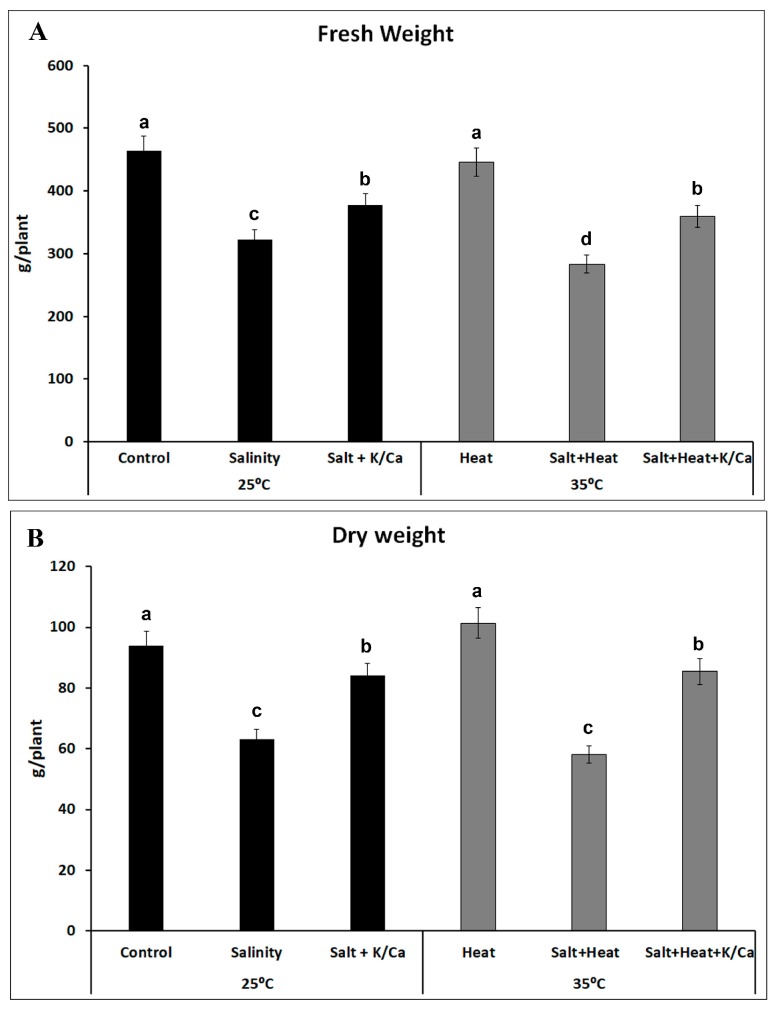
Fresh (**A**) and dry weight (**B**) of tomato plants grown under control, salinity (60 mM NaCl) or salinity and higher concentration of K^+^ and Ca^2+^ in the irrigation solution at optimal temperature (25 °C) or heat stress (35 °C). Data represent means ± SE (*n* = 6). Bars with different letters within each panel represent data with significant differences at *p* < 0.05 (Turkey HSD).

In arid and semi-arid regions, the scarcity of good quality water for agriculture necessitates the use of saline water, which has caused scientists to focus on studying the effect of salinity on plants. However, salinity is usually aggravated by the high temperatures that are found in these regions [5]. Our research group has previously shown that the study of the abiotic stressors in combination is essential, as plants showed a specific response to this combination that could not be deduced from the response to the individually-applied stressors alone [5,7]. As shown in Figure 1, the FW and DW of tomato plants were negatively affected by the application of salinity and salinity + heat, with DW reductions of 33% and 38%, respectively, as compared with control plants (Figure 1B). When the irrigation solution was complemented with increases in K^+^ and Ca^2+^, the effects of salinity and the combination of salinity and heat were diminished, obtaining plants with less biomass than control plants, but with reductions of only about 10% (Figure 1B). Thus, plants grown under salinity or under the combination of salinity and heat but with higher concentrations of K^+^ and Ca^2+^ in their nutrient solution increased their DW by 33% and 47% respectively, as compared to plants grown under these stressors and irrigated with the normal Hoagland solution. Capula-Rodriguez, et al. [38] highlighted the importance of the use of higher levels of K^+^ and Ca^2+^ in the irrigation solution for the mitigation of the effect of the combination of multiple stressors (salinity, alkalinity, and boron) in tomato plants, thus supporting our results. These authors observed that plants grown under the combination of high alkalinity, salinity, and an excess of boron showed improved growth when supplemented with greater concentrations of Ca^2+^ and K^+^, which was related to enhanced phosphorous concentration, maintenance of chlorophyll *a* concentration, and/or the partial restoration of the uptake of other nutrients under these stress conditions. 

**Figure 2:**
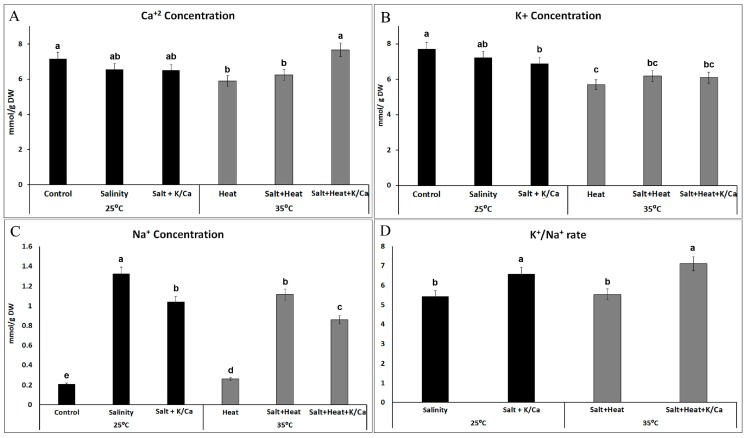
Ca^2+^, K^+^ and Na^+^ concentration (**A**–**C**) and K^+^/Na^+^ ratio (**D**) in tomato leaves grown under control, salinity (60 mM NaCl) or salinity combined with higher K^+^ and Ca^2+^ concentration in the irrigation solution under optimal temperature (25 °C) or under heat stress (35 °C). Values represent means ± SE (*n* = 6). Bars with different letters represent data with significant differences at *p* < 0.05 (Tukey HSD).

In general, salinity problems have been related to an excess of NaCl in the irrigation water, which causes Na^+^ toxicity in the plants, modifies the absorption of K^+^ and other nutrients by the roots, and exerts severe toxic effects on genes and enzymes, causing detrimental alterations to the plant’s metabolism [39,40,41]. Therefore, it is vital for plants to re-establish cellular ionic homeostasis to maintain correct metabolic functioning and growth. Our ionic analysis in tomato leaves detected that heat, and the combination of salinity and heat, reduced the endogenous concentration of Ca^2+^ and K^+^ (Figure 2A,B). Ca^2+^ was reduced by about 18% by heat and the combination of salinity and heat (Figure 2A), whereas K^+^ was reduced by these stressors by about 23% (Figure 2B), as compared to plants grown under optimal conditions (control plants). However, when salinity and heat were applied jointly, and the irrigation solution was supplemented with higher concentrations of K^+^ and Ca^2+^, the endogenous Ca^2+^ concentration obtained for the leaves was similar to control plants. Salinity did not induce significant changes in the leaves’ endogenous concentration of Ca^2+^ or K^+^ under any temperature treatment. However, surprisingly, when plants were grown under salinity and irrigated with higher K^+^ and Ca^2+^ concentrations, the endogenous concentration obtained for K^+^ was significantly lower than that obtained under control conditions, with a reduction of 12% (Figure 2B). 

The irrigation of plants with a nutrient solution enriched in K^+^ and Ca^2+^ and grown under a combination of stressors did not induce significant changes in the K^+^ concentration as compared to plants irrigated with the normal Hoagland solution under the same environmental conditions (Figure 2B). These data coincided with those previously observed in a study by Martinez et al., where the combination of salinity and heat caused an inhibition of K^+^ uptake. These reductions could be due to the antagonistic effect of K^+^ with Na^+^ [42]. In order to confirm Na^+^ and K^+^ homeostasis in our experiments, the endogenous Na^+^ concentration of leaves was studied (Figure 2C). 

As expected, the Na^+^ concentration increased significantly in those treatments where 60 mM NaCl was supplied. The salinity treatment had the highest Na^+^ concentration, and the application of salinity combined with heat induced a significantly lower Na^+^ accumulation in tomato leaves, with a reduction of 16% compared to salinity alone (Figure 2C). Also, the plants that received a nutrient solution enriched with K^+^ and Ca^2+^ and grown under stress conditions showed a lower Na^+^ accumulation in the leaves (~22% less) as compared with plants grown under the same conditions but irrigated with the normal Hoagland solution. Thus, our results indicated that endogenous concentrations of Na^+^ and K^+^ were lower when plants were irrigated with a nutrient solution enriched with K^+^ and Ca^2+^ under abiotic stress. The K^+^/Na^+^ ratio has been extensively related to the salt tolerance in plants [43]. In this sense, our experiments showed that this ratio was higher in those plants that were irrigated with higher concentration of K^+^ and Ca^2+^ (Figure 2D) under salinity or under the combination of salinity and heat, which could be related with plants that have an increased tolerance to these stressors. In this light, we found a positive correlation between biomass production and the K^+^/Na^+^ ratio in plants irrigated with higher K^+^ and Ca^2+^ and grown under salinity (DW_[Salt+K^+^/Ca^2+^]_ – K^+^/Na^+^ ratio_[Salt+K^+^/Ca^2+^]_, *r* = 0.971***) or the salinity and heat combination (DW_[Salt+Heat+K^+^/Ca^2+^]_ – K^+^/Na^+^ ratio_[Salt+Heat+K^+^/Ca^2+^]_, *r* = 0.811***). It has been postulated that some compounds (i.e., osmoprotectants and antioxidant compounds, among others) exert a regulatory function, maintaining a cytosolic K^+^ concentration by preventing NaCl-induced K^+^ leakage from the cell [12,14]. For example, K^+^ has been reported to result in the accumulation of osmolytes and augmentation of antioxidant components in the plants exposed to water and salt stress [44]. Thus, our results may indicate that an increase in K^+^ concentration in the nutrient solution may have an influence on the concentration of antioxidants and other beneficial compounds in plants, and therefore on the maintenance of the K^+^/Na^+^ homeostasis in plants. The increase in K^+^ and Ca^2+^ concentrations in the irrigation solution improved tomato salinity tolerance under both optimal temperature and heat stress. The most aggressive stress treatment for tomato plants was salinity and heat; however, an increase in K^+^ and Ca^2+^ concentration in the irrigation solution induced a higher recovery of these plants under salinity and heat stress. 

**Figure 3:**
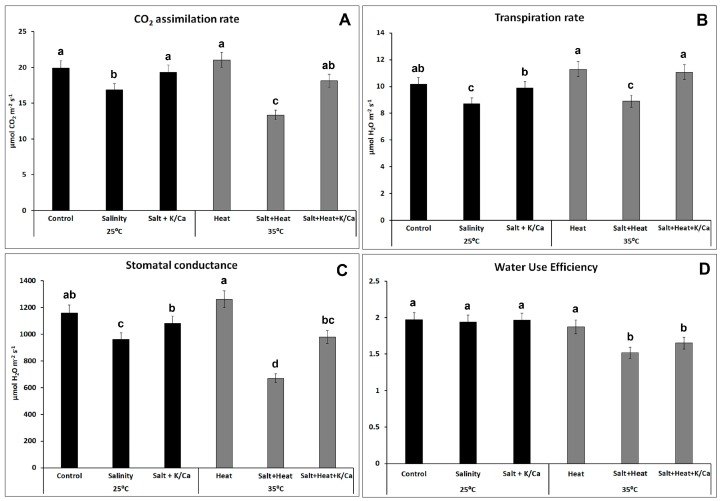
Photosynthetic parameters in tomato leaves grown under control, salinity (60 mM NaCl or salinity combined with higher K^+^ and Ca^2+^ concentration in the irrigation solution at optimal temperature (25 °C) or heat stress (35 °C). Values are means ± SE (*n* = 6). Bars with different letters within each panel represent data with significant differences at *p* < 0.05 (Tukey HSD).

At the end of the experimental period and before the plants were harvested, photosynthetic parameters, such as CO_2_ assimilation rate, stomatal conductance, transpiration rate, and water use efficiency (calculated from the CO_2_ assimilated versus the water transpired) were measured (Figure 3). Changes in these parameters are usually good indicators of stress in plants and they are directly correlated with plant growth. CO_2_ assimilation rate (Figure 3A), transpiration rate (Figure 3B), and stomatal conductance (Figure 3C) had a similar behavior in our experiments with plants under stress conditions. As compared to control conditions, the salinity and salinity + heat treatments resulted in a significant reduction of those parameters with respect to control plants, with salinity + heat being more aggressive than salinity itself. These data were consistent with the findings by Pinero, et al. [45] and Rivero et al. [7], who reported that both the salinity and salinity + heat treatments led to a reduction in stomatal conductance, reducing the plant’s ability to supply CO_2_ to the photosynthetic apparatus, thus decreasing the CO_2_ assimilation rate under these stress conditions. On the contrary, it has also been observed that heat stress alone can favor a greater transpiration rate, in order to reduce leaf temperature, which may lead to an increase in the CO_2_ assimilation rate. The increase described for those parameters was also observed in these experiments, which were therefore in agreement with the results found by Rivero et al. [7]. Interestingly, when plants were grown under salinity or salinity + heat but supplemented with a nutrient solution enriched with K^+^ and Ca^2+^, CO_2_ assimilation, transpiration rate, and stomatal conductance partially recovered to values similar or closer to control levels (Figure 3A–C). These photosynthetic parameters are closely related to growth rate in plants, and our results indicate a positive correlation among them, where a reduction in plant growth (Figure 1) was directly related with an inhibition of these photosynthetic parameters, and the recovery of these by an extra supply of the plants with K^+^ and Ca^2+^ implied better growth under abiotic stress conditions. Abass et al. [44] and Bohra and Doerffling [46] found similar results in maize plants and rice, respectively, through the addition of K^+^ to a saline soil. Both studies concluded that the addition of K^+^ significantly alleviated the harmful effects of salinity, by improving plant growth and gas exchange parameters. 

In addition to K^+^, Ca^2+^ also plays an important role in the plant cell. Among its numerous cell functions, Ca^2+^ contributes to the cell’s signaling processes and helps with the detoxification of ROS [18,47,48]. Bhattacharjee [49] observed that the addition of Ca^2+^ helped to protect against heat-induced oxidative damage. It is widely known that salinity and heat stress can enhance oxygen-induced cellular damage due to a high production of ROS [6,22,50,51], so the importance of enhancing the antioxidant defense plant system to cope with these stressors should be highlighted. In recent studies, it has been observed that plants subjected to different combinations of abiotic stressors accumulated large amount of ROS response transcripts, which defined the importance of the antioxidant machinery on the acclimation pathways during combined stressors [50,52]. Martinez et al. [6] showed that tomato plants subjected to a combination of salinity and high temperature had an overproduction of ROS that caused photosynthesis inhibition, breakdown of photosynthetic pigments, and plant growth reduction. However, although the study of the combined effect of two abiotic stressors has begun and there are many publications that demonstrate the importance of studying stressors in combination, there is little information about the effect of the nutrition management and its possible relationship with abiotic stress combination tolerance in plants. In light of this question, a detailed study of the plant’s oxidative metabolism was performed in our plants in order to verify a possible role of the extra supply of K^+^ and/or Ca^2+^ on the enhancement of the antioxidant defense pathways in plants. 

**Figure 4:**
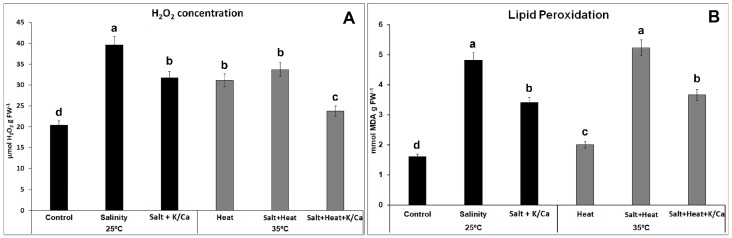
H_2_O_2_ concentration (**A**) and Lipid peroxidation rate, measured as MDA concentration (**B**) in tomato leaves grown under control, salinity (60 mM NaCl) or salinity combined with a higher concentration of K^+^ and Ca^2+^ in the irrigation solution at optimal temperature (25 °C) or under heat stress (35 °C). Values represent means ± SE (*n* = 6). Bars with different letters within eahc panel represent data with significant differences at *p* < 0.05 (Tuket HSD).

Under these environmental stress conditions, where water supply to the plant is being compromised (i.e., with regards to salinity, heat or both), the internal cellular CO_2_ concentration is restricted due to stomatal closure. This, together with the continuous incident sunlight to the plant, results in a transfer of excess electrons to molecular oxygen, generating a superoxide radical (O_2_^−^•), which is immediately detoxified to H_2_O_2_ by superoxide dismutases (SODs). In addition, the stress condition generates irreversible damage to the cellular membrane’s polyunsaturated fatty acids. Thus, two of the most common oxidative stress markers used to measure oxidative damage to the cells are H_2_O_2_ concentration and membrane lipid peroxidation, measured as the concentration of MDA derivatives. These two parameters were evaluated in our experiments with tomato plants (Figure 4). Any of the stress conditions used in our experiments resulted in a significant increase in the H_2_O_2_ concentration with respect to control plants, with the salinity treatment being the one with the highest concentration of this compound, which was found to be almost double the amount found in control plants. On the other hand, when plants were grown either under salinity or the combination of salinity and heat but irrigated with a nutrient solution enriched with K^+^ and Ca^2+^, the concentration of H_2_O_2_ was significantly lower than that found under the same environmental conditions but irrigated with the normal Hoagland solution. Thus, plants irrigated with higher K^+^ and Ca^2+^ and grown under salinity had 20% less H_2_O_2_ than those grown under salinity but irrigated with the normal nutrient solution. Interestingly, when salinity was combined with heat, the extra supply of K^+^ and Ca^2+^ in the nutrient solution resulted in a 30% reduction in H_2_O_2_ concentration. For the MDA content, the results obtained were similar, with the higher concentration of this compound found in plants under salinity and salinity + heat treatments and irrigated with the normal nutrient solution. Again, the addition of extra K^+^ and Ca^2+^ to the nutrient solution resulted in a significant reduction in the level of lipid peroxidation of these plants under these stress conditions. H_2_O_2_ accumulation and membrane lipid peroxidation leads to oxidative damage to the cells, inhibiting photosynthesis and inducing plant growth inhibition [7,28]. Our results showed that tomato plants grown under these treatments, where H_2_O_2_ was accumulated to higher concentrations (Figure 4A), showed a higher membrane lipid peroxidation (Figure 4B) and an significant impairment of the photosynthetic parameters (Figure 3), with the concomitant inhibition of plant growth.

**Figure 5:**
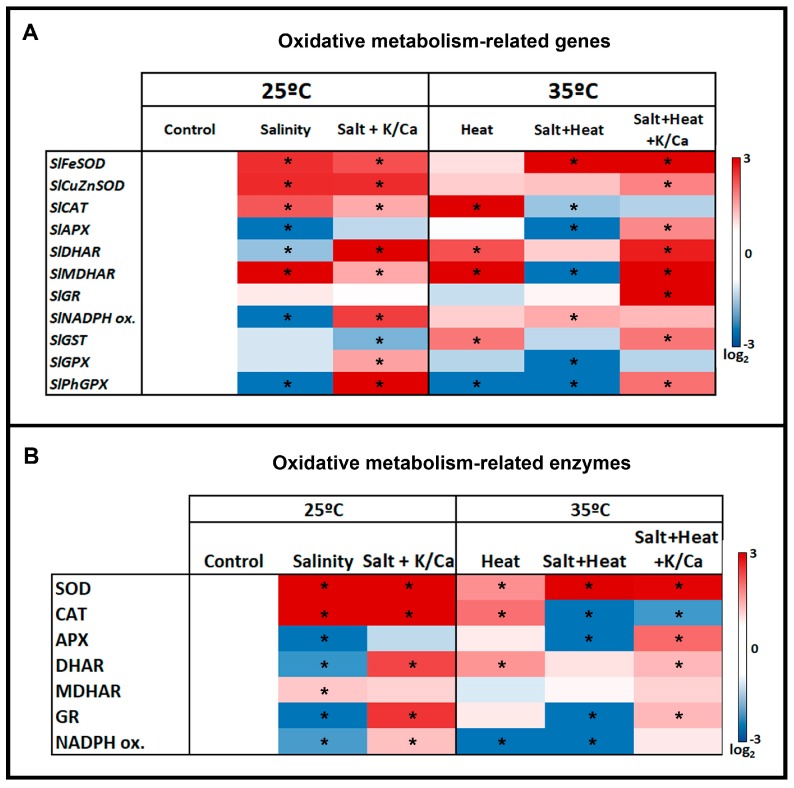
Oxidative metabolism-related gene expression (**A**) and enzymatic activities (**B**) in tomato leaves grown under control, salinity (60 mM NaCl) or salinity and higher concentration of K^+^ and Ca^+2^ in the irrigation solution at optimal temperature (25 °C) or heat stress (35 °C). Red color represents a higher relative expression or activity with respect to control plants and blue color represents a lower relative expression or activity. Scale is the log_2_ values of the expression after normalization with respect to control plants. Absolute values (gene expression and enzymatic activities) as well as log2 values can be found in Supporting Information Tables S2–S5, respectively. Asterisks are representative of significant differences with respect to control plants (*p* < 0.05).

The impairment of the cell antioxidant machinery under abiotic stress conditions can be caused by an overproduction of ROS, an inhibition of the antioxidant enzymes, or both. This inhibition may occur at transcriptional or post-transcriptional levels, or by post-translational modifications of the antioxidant proteins necessary for counteracting the deleterious effects of ROS. In order to assess these impairments, the expression of the main oxidative metabolism-related transcripts and the activity of the main antioxidant enzymes were measured under the different stress treatments used in our study (Figure 4A). After examining both heatmaps (Figure 5A,B) it is clear that tomato plants have a specific antioxidant response under the combination of salinity and heat, and that the response of the genes or enzymes cannot be deduced from the individual responses obtained under salinity or heat applied individually. These results confirm those obtained previously by Rivero et al. [7], Martinez et al. [5], and Martinez et al. [6], and highlight the importance of studying abiotic stressors in combination. The expression levels of the transcripts that code for Fe-SOD and Cu/Zn-SOD were upregulated under all the stress conditions (Figure 5A), and correlated well with the SOD activity found, with the exception of Cu/Zn-SOD activity in plants grown under the combination of salinity + heat and irrigated with the nutrient solution enriched with K^+^ and Ca^2+^, in which case this enzyme was inhibited.

The first cellular line of defense for ROS detoxification is constituted by SODs, which detoxify superoxide radicals into H_2_O_2_. In this sense, the over-production of H_2_O_2_ observed under the different stress treatments applied in our experiments was due to an over-expression of Fe-SOD and Cu/Zn-SOD encoding transcripts (Figure 4A), with a concomitant increase in SOD activity. H_2_O_2_ levels found in the stress treatments of plants supplemented with the K^+^/Ca^2+^ enriched nutrient solution were lower than those of plants grown with the normal nutrient solution under the same stress conditions, which may be due to: (1) a lower production of this compound in these treatments, due to the beneficial effects of the extra supply of K^+^ and Ca^2+^ under stress conditions, or (2) a more effective H_2_O_2_ detoxification driven by an overexpression of Fe-SOD encoding transcripts (Figure 5A), with a concomitant increase in SOD activity (Figure 5B, Supplementary Table S4) under these conditions. H_2_O_2_ accumulation also has a deleterious effect in plant cells, which correlated well with the biomass production obtained in our experiments under all the stress treatments used (Figure 1). Therefore, an effective detoxification system for H_2_O_2_ must work in coordination in order to avoid the cellular accumulation of this compound. In this sense, CAT and the ascorbate–glutathione cycle act together to detoxify H_2_O_2_ from the cells to H_2_O. In our experiments, CAT encoding transcripts were upregulated under salinity, independently of the nutrient solution used. Also, this transcript was upregulated under heat stress, whereas in the salinity + heat treatment it was downregulated. These results correlate well with the CAT activity obtained under these conditions, concluding that the extra supply of K^+^ and Ca^2+^ did not have any effect on the transcription and activity of this enzyme under stress conditions, but the combination of salinity and heat induced the inhibition of its transcription.

The ascorbate–glutathione (AsA–GSH) cycle acts in parallel to CAT in the effective detoxification of H_2_O_2_. In our experiments, the transcription levels of the different transcripts that codify for the antioxidant enzymes from the AsA–GSH cycle were differently affected depending on the stress treatment and the irrigation solution used. Thus, salinity stress downregulated the transcripts that codify for APX, DHAR, and NADPH oxidase, with the concomitant inhibition of their related enzymes. Interestingly, the supplementation of plants with a nutrient solution enriched with K^+^ and Ca^2+^ reverted the regulation observed for DHAR and NADPH oxidase under salinity, inducing the upregulation of those genes as compared to control plants. However, the activity of DHAR and NADPH oxidase did not show significant differences with respect to control plants. MDHAR transcripts were upregulated and the activity of this enzyme was also higher under salinity as compared to control plants, independently of the irrigation solution used. Heat stress induced an upregulation of all the transcripts belonging to the AsA–GSH pathway, except for *SlAPX* and *SlGR,* which did not show significant differences with respect to control plants. When salinity and heat stress were applied jointly, a general downregulation of the transcripts codifying for the enzymes belonging to the AsA–GSH pathway was observed, with the concomitant inhibition of their enzymatic activities. However, the feeding of the plants with the nutrient solution enriched with K^+^ and Ca^2+^ reverted the expression profile observed under the salinity and heat combination for most of the genes, with an upregulation pattern observed in all of them as compared to the control plants, which correlated with the activities measured for these enzymes. The inhibition of one or more of these enzymes in plants grown under salinity or under salinity + heat may explain the high H_2_O_2_ concentration found under these treatments, where the detoxification of this compound was not as efficient as compared to control plants. This resulted in the accumulation of this compound, oxidative damage, and plant growth inhibition [7,28]. On the other hand, the supplementation of plants with a nutrient solution containing a higher concentration of K^+^ and Ca^2+^ seemed to induce the transcription of all the genes involved in the efficient detoxification of H_2_O_2_ (i.e., *SlAPX, SlDHAR, SlMDHAR, SlGR, SlNADPH* oxidase), significantly reducing the oxidative damage observed in plants grown under the combination of salinity + heat, and the activation of stress-related genes implied in the signaling mechanisms for ROS detoxification [10]. Thus, K^+^ and Ca^2+^ may contribute to the maintenance of the cellular redox homeostasis, protecting photosynthesis and electron transport systems and increasing plant tolerance to abiotic stressors. 

GST, PhGPX, and GPX catalyze the GSH-dependent reduction of H_2_O_2_ and organic peroxides, including lipid peroxides to H_2_O or alcohols [44]. In our experiments, the expression of the transcripts *SlGST*, *SlPhGPX,* and *SlGPX* were determined in order to compare them with the level of lipid peroxidation observed under the different treatments and to verify if a supplementation of the plant irrigation solution with K^+^ and Ca^2+^ might have any effect on the transcription of these genes. Our results showed that under salinity and salinity + heat, these three genes were downregulated with respect to control plants. However, in plants fed with a nutrient solution enriched with K^+^ and Ca^2+^, an upregulation of the *SlGPX* and *SlPhPGX* (under salinity) and of the *SlGST* and *SlPhGPX* (under salinity + heat) was observed. These results correlated well with the data obtained for lipid peroxidation level in these plants (Figure 4B), where it was observed that K^+^ and Ca^2+^ used in higher than recommended concentrations lowered the lipid peroxide level under salinity and salinity + heat as compared with plants irrigated with the normal nutrient solution and grown under the same stress conditions. In this sense, our results may indicate that K^+^ and Ca^2+^ could play a role in the transcriptional regulation of the genes that codify for the enzymes involved in the reduction of the H_2_O_2_ and, consequently, may also play a role in the protection of membrane lipids from peroxidation in plants under abiotic stress conditions. 

## 4. Conclusions

The results obtained in this work highlight the complexity and importance of using the correct supplementation in the plant irrigation solution. We have demonstrated that the plant’s tolerance to salinity—and more importantly, to the combination of salinity and heat—can be improved by the proper enrichment of the nutrient solution with K^+^ and Ca^2+^. These nutrients, used in higher concentrations than recommended, helped to control oxidative damage through the transcriptional regulation of the main enzymes implicated in ROS detoxification and the recovery of membrane lipid peroxides. Consequently, a better performance of the photosynthetic apparatus was maintained, leading to optimal growth rates. Our study highlights the importance of plant nutrition in the signaling processes that control plant tolerance responses to abiotic stress combination. However, more studies are needed in order to fully elucidate the role of K^+^ and Ca^2+^ in these signaling mechanisms.

## Figures and Tables

**Table 1:** Nutritional and environmental temperature treatments applied in the preliminary experiment to the tomato plants in order to select the most significant nutritional treatment for further experiments.

Growth Chamber	Treatments	[NaCl]	[K^+^]	[Ca^2+^]
Chamber A (25 °C)	Control	0 mM	7 mM	4 mM
	Salinity	60 mM	7 mM	4 mM
	Salinity + T1	60 mM	8 mM	4.7 mM
	Salinity + T2	60 mM	9.8 mM	5.6 mM
	Salinity + T3	60 mM	11 mM	6.5 mM
Chamber B (35 °C)	Heat	0 mM	7 mM	4 mM
	Salinity + Heat	60 mM	7 mM	4 mM
	Salinity + Heat + T1	60 mM	8 mM	4.7 mM
	Salinity + Heat + T2	60 mM	9.8 mM	5.6 mM
	Salinity + Heat + T3	60 mM	11 mM	6.5 mM

**Table 2:** Nutritional and environmental temperature treatments applied to tomato plants under greenhouse conditions for the physiological, biochemical and molecular determinations.

Greenhouse	Treatments	[NaCl]	[K^+^]	[Ca^2+^]
Greenhouse A (25 °C)	Control	0 mM	7 mM	4 mM
	Salinity	60 mM	7 mM	4 mM
	Salinity + K/Ca	60 mM	9.8 mM	5.6 mM
Greenhouse B (35 °C)	Heat	0 mM	7 mM	4 mM
	Salinity + Heat	60 mM	7 mM	4 mM
	Salinity + Heat + K/Ca	60 mM	9.8 mM	5.6 mM

## Supplementary Materials

The following are available online at https://www.mdpi.com/2076-3921/8/4/81/s1, Table S1: Nutritional composition of the Hoagland solution used for tomato plants growth, Table S2: Primers used for the quantification of the expression levels of the oxidative metabolism-related transcripts by qPCR, Table S3: Relative expression values of the oxidative metabolism-related transcripts. Values were normalized against control samples and log2 was calculated and shown. Values are means of *n* = 3, Table S4: Absolute activities of the oxidative metabolism-related enzymes. Values obtained were normalized using soluble protein content of each sample and treatment. Values are the means ± SE (*n* = 3), Table S5: Log_2_ of the oxidative metabolism-related enzymes. Values obtained in Table S3 were normalized against control and log2 was calculated, Figure S1. Fresh weight (FW) of tomato plants obtained at the end of the preliminary experiment (see Materials and Methods section).
