# Supplementary material for: Amelioration of the Oxidative Stress Generated by Simple or Combined Abiotic Stress through the K+ and Ca2+ Supplementation in Tomato Plants
Source: Antioxidants (Basel). 2019 Mar 30;8(4):81. doi: 10.3390/antiox8040081 (PMC6523471; doi:10.3390/antiox8040081)

Supplementary Table S1. Nutritional composition of the Hoagland solution used for tomato plants growth.

	Concentration stock solution (mM)	Grams of compound used (g)	Volume of stock solution per liter for full strength (mL)	Element	Final concentration of each element
**Compound**	**Macronutrients**				
**KNO_3**	1,000	101.10	6.0	N	14 mM
**$Ca(NO_3)_2 \cdot 4H_2O$**	1,000	236.16	4.0	**K**	**7 mM**
**$NH_4H_2PO_4$**	1,000	115.08	2.0	**Ca**	**4 mM**
**$MgSO_4 \cdot 7H_2O$**	1,000	246.48	1.0	P	1 mM
				S	1 mM
				Mg	1 mM
	**Micronutrients**				
**KCl**	50 mM	3.728	1.0	Cl	50 µM
**H_3BO_3**	25 mM	1.546	1.0	B	25 µM
**$MnSO_4 \cdot H_2O$**	2 mM	0.338	1.0	Mn	2.0 µM
**$ZnSO_4 \cdot 7H_2O$**	2 mM	0.575	1.0	Zn	2.0 µM
**$CuSO_4 \cdot 5H_2O$**	0.5 mM	0.125	1.0	Cu	0.5 µM
**$(NH_4)_6Mo_7O2_4 \cdot 4H_2O$**	0.5 mM	0.088	1.0	Mo	0.5 µM
**Fe-EDTA**		18.6	1.0	Fe	20 µM

Supplementary Table S2. Primers used for the quantification of the expression levels of the oxidative metabolism-related transcripts by qPCR.

**Gen**	**Accession (SGN)**	**Forward**	**Reverse**
SlFe-SOD	Solyc06g048410	taaatagagactttggttcc	tatatttgcctcttaaccct
SlCu/Zn-SOD	Solyc11g066390	ggccaatctttgacccttta	agtccaggagcaagtccagt
SlCAT1	Solyc12g094620	tgatcgcgagaagatacctg	cttccacgttcatggacaac
SlcAPX	Solyc06g005160	tctgaattgggatttgctga	cgtctaacgtagctgccaaa
SlDHAR1	Solyc05g054760	aggtggctcttggacacttc	cttcagccttggttttctgg
SlMDHAR1	Solyc08g081530	caagggtttcggttccttct	ctgcatttcctcctccaact
SlGR1	Solyc09g091840	ttggtggaacgtgtgttctt	tctcattcacttcccatcca
SlGST	Solyc01g086680	tactcgtttttgggctcgtt	caccgattcaactccctctg
SlGPX	Solyc08g080940	acggagcaagcgacaattgacaac	cgattgattcaccgcaaagctcgt
SlNADPH ox.	Solyc08g081690	agggaatgatagagcgtcg	catcgtcattggacttggc
SlPhGPX	Solyc06g073460	tggcttgagcactacaggtg	tttcgtaggcagggaagaag

Supplementary Table S3. Relative expression values of the oxidative metabolism-related transcripts. Values were normalized against control samples and log2 was calculated and shown. Values are means of n = 3.

	25ºC			35ºC		
	Control	Salinity	Sal+K/Ca	Heat	Sal+Heat	Sal+Heat +K/Ca
SlFeSOD	0	2.42158333	2.05563333	0.39758333	3.48083333	4.06833333
SlCuZnSOD	0	2.461	2.456	0.59833333	0.74075	1.471
SlCAT	0	1.9568	1.02815	5.39906667	-0.8524	-0.6534
SlAPX	0	-2.03426667	-0.56526667	-0.02673333	-3.2169	1.4108
SlDHAR	0	-0.907	4.28613333	2.04283333	0.589	2.60916667
SlMDHAR	0	3.9126	1.0466	3.27356667	-4.47423333	3.4462
SlGR	0	0.26366667	0.02916667	-0.48366667	0.11533333	5.75166667
SlNADPH ox.	0	-3.24581549	2.25544873	0.58474158	1.02254785	0.84578553
SlGST	0	-0.36483333	-1.10666667	1.62233333	-0.56686667	1.62416667
SlGPX	0	-0.36483333	1.10666667	-0.62233333	-2.56686667	-0.62416667
SlPhGPX	0	-2.28666667	3.4692	-2.08916667	-3.78433333	1.68366667

Supplementary Table S4. Absolute activities of the oxidative metabolism-related enzymes. Values obtained were normalized using soluble protein content of each sample and treatment. Values are the means ±SE (n=3).

	25ºC			35ºC		
	Control	Salinity	Sal+K/Ca	Heat	Sal+Heat	Sal+Heat +K/Ca
SOD	3.87	7.88397761	14.8276913	5.25738401	12.5794411	7.5644212
CAT	11.57	24.7008567	25.0222683	17.1339269	60.7050015	6.91760787
APX	22.39	7.769575	18.5499068	23.758835	7.01665118	33.596195
DHAR	17.03	9.97439408	28.1130161	22.8185266	18.5127943	20.8190521
MDHAR	11.25	13.1362593	12.7675169	10.0775882	11.5005558	12.7675169
GR	29.65	6.12922478	51.1977443	31.5015336	14.001947	36.1420557
NADPH ox.	9.36	5.6815063	11.1636462	2.62508918	3.94031227	9.99855759

Supplementary Table S5. Log_2 of the oxidative metabolism-related enzymes. Values obtained in Supplementary Table S4 were normalized against control and log2 was calculated.

	25ºC			35ºC		
	Control	Salinity	Sal+K/Ca	Heat	Sal+Heat	Sal+Heat +K/Ca
SOD	0	1.02659011	1.93788852	0.44201155	1.70066236	0.96689613
CAT	0	1.09417221	1.11282371	0.56646698	-2.39142652	-0.74204372
APX	0	-1.52694694	-0.27144259	0.08560957	-1.67399998	0.58544332
DHAR	0	-0.77177733	0.72315981	0.42212721	0.12044424	0.28982595
MDHAR	0	0.22362951	0.18255297	-0.15877459	0.03177858	0.18255297
GR	0	-2.27425559	0.78804814	0.08738996	-1.08240465	0.28564646
NADPH ox.	0	-0.72023506	0.25422787	-1.83414209	-1.24819856	0.09521145

Supplementary Figure S1. Fresh weight (FW) of tomato plants obtained at the end of the preliminary experiment (see Materials and Methods section)

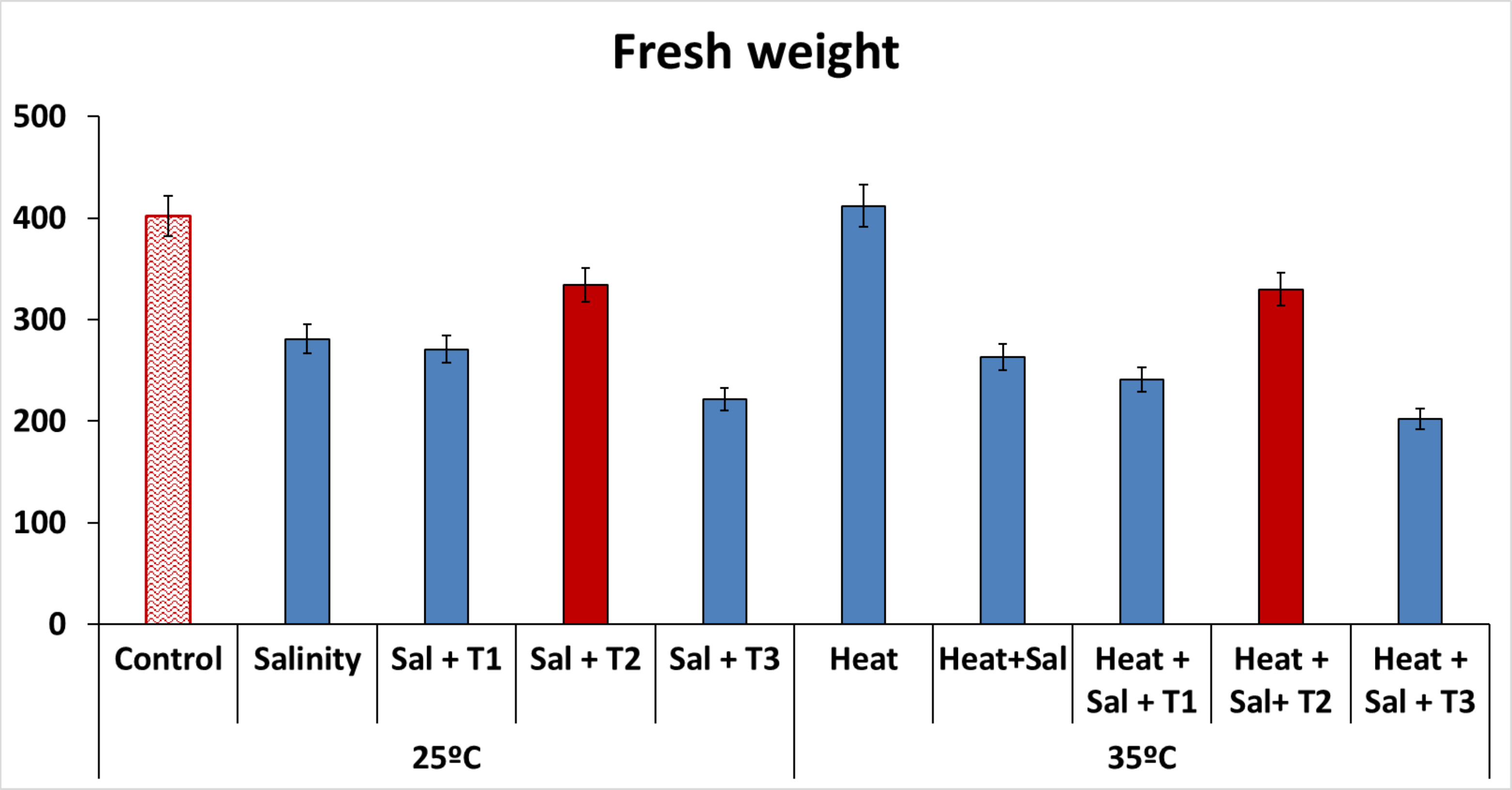

Supplementary Photograph S1. Photograph of the experimental design in one of the greenhouses used for our experiments.

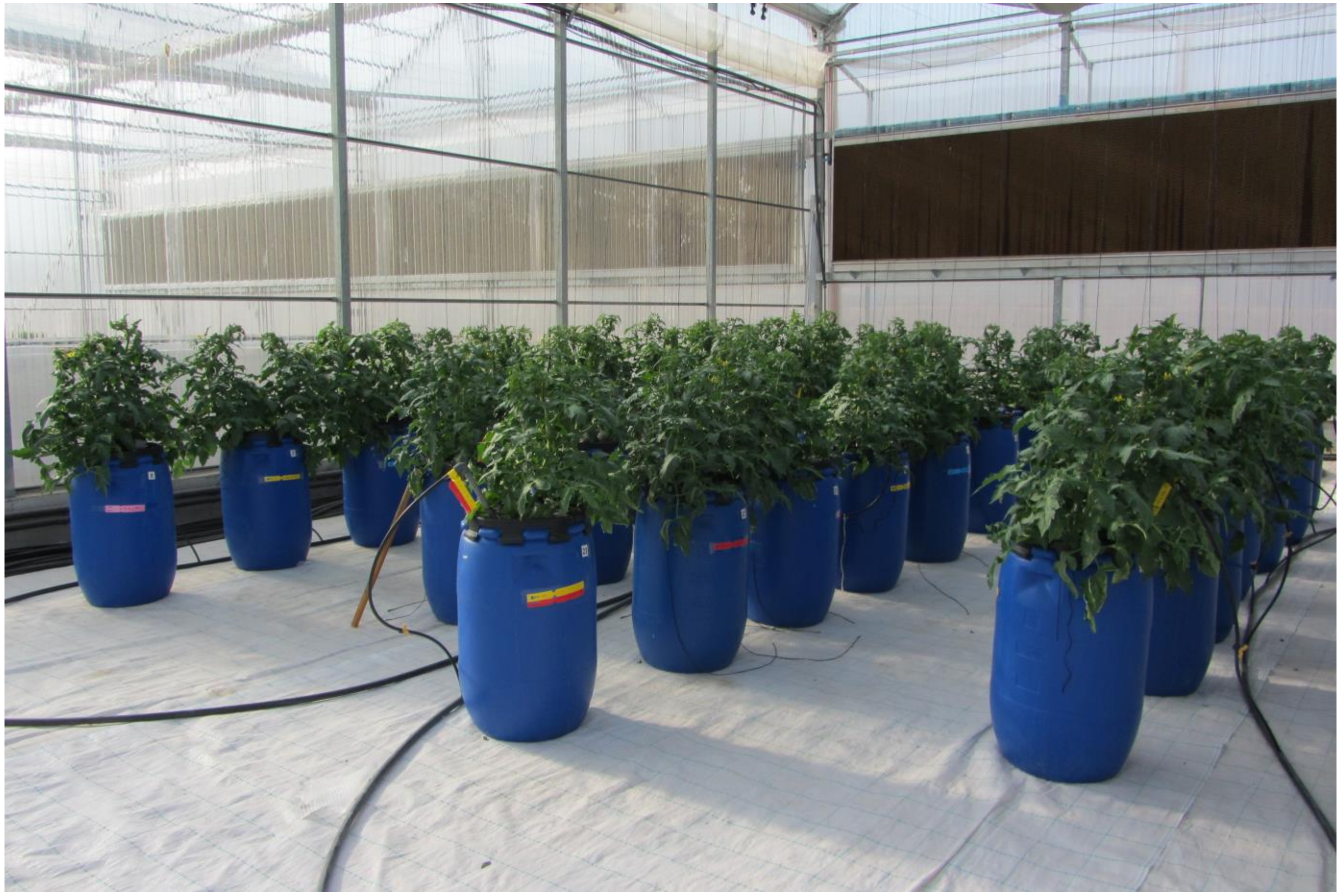

Supplement: Supplementary file 1 [file antioxidants-08-00081-s001.pdf]
